# Developing Indicators to Evaluate Instructor Management of Sports Centers for the People With Disabilities Based on Universal Design Principles in South Korea

**DOI:** 10.3389/fpubh.2022.871468

**Published:** 2022-05-25

**Authors:** Eunsurk Yi, Jongseob Shin, Ahra Oh

**Affiliations:** Department of Exercise Rehabilitation and Welfare, Gachon University, Incheon, South Korea

**Keywords:** universal design, disability, Delphi technique, instructor management, sports centers

## Abstract

This study was conducted to develop evaluation indicators for instructor-led management of sports centers for the disabled using universal design (UD) principles in South Korea. These indicators have been developed through Delphi technique to identify the effectiveness of an instructor's management skills. There were 11 documents related to UD used in the literature review, and seven were related to the evaluation index. Through reading and analyzing the relevant contents of the collected literature and many rounds of the Delphi technique, we selected the method and criteria for deriving the evaluation index. In this study, we developed a method that constitutes an evaluation index. The index comprises one evaluation criterion and four evaluation indices. First, for the sub-items of the “recruitment” category, four principles of UD and one supplementary principle of product performance program (PPP) were applied to create items for the evaluation index. Second, the sub-items of the “education” category comprise three evaluation criteria and 10 evaluation indicators. These were applied to the fourth principle of UD and the first and second by-supplementary principles of PPP. The third category, “welfare,” comprised two evaluation criteria and six evaluation indices, and the first by-supplementary principle of PPP was applied to the evaluation indices. The index created for evaluating instructors in sports centers using the method elucidated in this study was adequately reliable. Following a similar method, more evaluation indicators should be developed for evaluations of other functions (such as programs, public relations, safety, and finance) based on the principles of UD.

## Introduction

Universal Design (UD) was introduced in the 1970s by Ronald Mace, an American architect and product designer with disabilities (1). UD considers human diversity in the design of products and spaces and follows the design of architects and designers (2). In addition, UD aims to create not only a value system that informs the design of environments but also products that meet the needs of all users (3).

UD is already in use in various fields in our society. It is utilized not only in building centers but also in education, IT, and medicine. In particular, in most developed countries, UD is employed in fields that regularly include people with disabilities and members of the general public to provide spaces accessible and practical for everyone; they do not distinguish between spaces used by people with disabilities and those used by others. In other words, in UD spaces, the whole room, building, complex, or facility seamlessly accommodates all members of a diverse population rather than designating distinct, separate spaces intended for individuals with particular disabilities (1, 4, 5).

In most developed countries, UD environments are being created for use by the disabled and non-disabled together. Likewise, Korean governments are also aware of the importance of UD and are planning to build and expand a sports center for the disabled incorporating UD. It has planned to build 30 centers in 2019, 23 centers in 2020, and 30 centers in 2021. In total, the number of sports centers for the disabled will be expanded to 150 by 2025 (6).

In this background, we aimed to create a continuous legacy along with the successful hosting of the 2018 Pyeongchang Winter Paralympics. The name of the Bandabi Sports Center was created in honor of “Bandabi,” the mascot of the 2018 Pyeongchang Winter Paralympics in support of guarantees of the priority rights for the disabled and to support the selection of regional customized models suitable for regional characteristics among gymnasiums, swimming pools, and sports specialized models as integrated sports centers used by non-disabled people. In particular, the Bandabi Sports Center was built as a UD because Rep. Kim emphasized it should be built as a national sports center for both the disabled and the general public without emphasizing the disabled (7).

Based on the UD principle, recruiting and managing instructors who are able to operate sports programs for patrons with disabilities is important for effectively distinguishing gyms that accommodate users with disabilities from what previously were “regular” gyms. In other words, a UD sports facility for the disabled can be considered “well-operated” when all of the instructors recognize and guide the concept of UD.

Over the years, many studies related to UD have been conducted, including some research combining UD with sports (8–10), such as studies of UD-related learning (11–14) and UD-related program and device development (15, 16). Research on spatial architecture incorporating UD (17, 18) and one study on the concept establishment of UD (19) have also been conducted. In addition, research on evaluation indicators for the management of sports instructors included a study on instructorship education ability evaluation (20, 21), a study on policy development (22, 23), and a study on leadership role based on the development of leadership education programs (24). Their research presents various discussions, including the reasons for grafting UD, the advantages and disadvantages of UD, and improvement measures. However, there is a scarce study in the development of evaluation indicators for the management of instructors of sports centers for the disabled incorporating UD.

We found one paper that was contextually similar to the present study: Watchorn et al. (25) considered occupations for inclusion in the discourse about architectural environments incorporating UD. The researchers combined quantitative and qualitative methods to present discourses on the jobs that are necessary and the people who are suitable for the UD occupations. Although many UD environments are incorporated into our society, the researchers concluded that such occupations should be created because there are not yet any suitable people for the various types of jobs necessary to operate UD centers and businesses. However, it can be seen that there are some differences between the available literature and this study: we aimed to clarify how to assess and manage instructors who work with all types of users, able-bodied or otherwise, in UD sports centers. Therefore, in this study, it is time to develop evaluation indicators for instructor management of sports centers for the disabled based on UD in Korea.

Therefore, the purpose was to develop an evaluation index that can evaluate the suitability of hiring and managing instructors in UD-based sports centers for the disabled based on the analysis results. This study examined the opinions on what UD elements should be employed and managed by instructors of sports centers for the disabled based on the UD principle through Delphi technique were collected and analyzed.

## Materials and Methods

### Research Procedure

The indicators for the evaluation of sports instructors at all centers for disabled people to which UD was applied were derived over a total of four stages. As a first step, the extent of incorporation of the UD elements and the method of deriving evaluation indices were determined through literature research. In the second step, 17 chosen experts were subjected to the first modified Delphi technique. Among the seven principles of UD and three supplementary principles of product performance program (PPP), three elements to be reflected in the evaluation index, evaluation criteria, and evaluation index items were extracted and classified. The Delphi panel configuration is shown in Table 1.

**Table 1:** Delphi panel configuration.

**Configuration**	**Affiliation organization**	**Position**
Disabled sports specialist	Seoul Aquatic Rehabilitation Center	Center director
	Jeongjeong Hall	Sports team instructor for the disabled
	Gwangju Metropolitan City Disabled National Sports Center	Sports team instructor for the disabled
	Goyang City Rehabilitation Sports Center	Center director
	Seongnam City Hanmaeum Welfare Center	Sports team instructor for the disabled
	Jecheon City Eoullim Sports Center	Sports team instructor for the disabled
	Asan National Sports Center for the Disabled	Sports team instructor for the disabled
	Jeonju Eoullim National Sports Center	Sports team instructor for the disabled
	Gwangyang National Sports Center for the Disabled	Center director
	Gumi City Gymnasium for the Disabled	Sports team instructor for the disabled
	Changwon City Gomduri National Sports Center	Sports team instructor for the disabled
	Chuncheon Disabled Sports Center	Sports team instructor for the disabled
	Seoul Gomduri Sports Center	Sports team instructor for the disabled
Senior sports specialist	Seo-gu Senior Welfare Center	Sports team instructor for the senior
	Yeonsu Senior Welfare Center	Sports team instructor for the senior
	Michuhol Senior Welfare Center	Sports team instructor for the senior
	Yeonsu Senior Welfare Center	Center director

**Figure 1:**
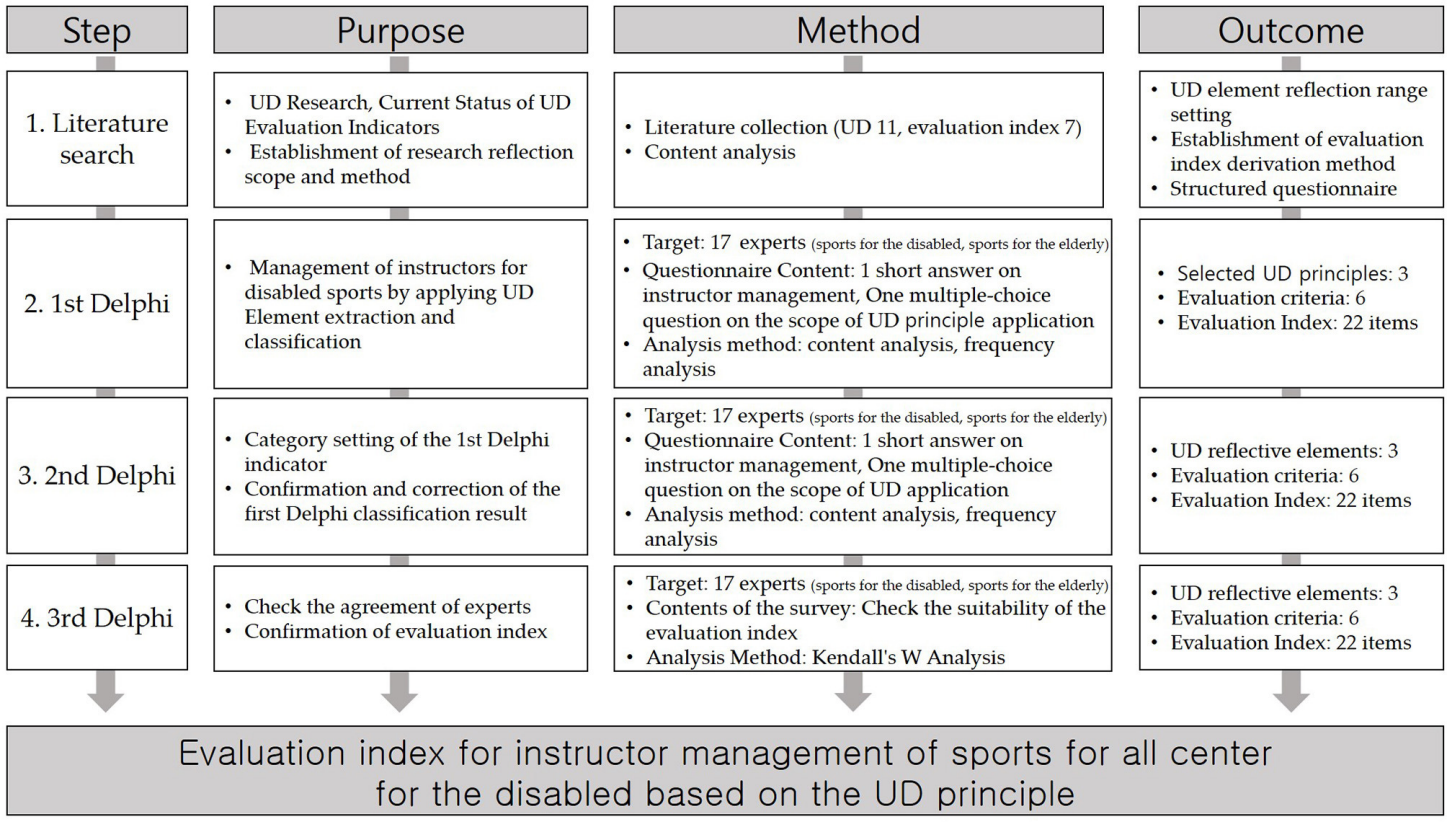
Research Flow.

As the third step, the second modified Delphi technique was carried out to confirm and revise the reflection factors of UD, evaluation criteria, and classification results of evaluation indicators. The final methodology step was to confirm the degree of agreement among experts regarding the evaluation categories, the criteria for selection, and index contents. This was done by using Kendall's Coefficient of Concordance W (Kendall's W) analysis and intraclass correlation coefficients (ICC). The detailed research procedure is shown in Figure 1.

### Developing Evaluation Indicators

Eleven documents related to UD were used in the literature review, and seven documents related to the construction of the evaluation index. These documents were carefully analyzed, the results helped in setting the scope of evaluation indicators to be reflected in this study. The scope of UD was applied using the Center for Universal Design (1997) and the proposed Universal Design Product Performance Program (PPP) (26). The modified Delphi technique was assessed as suitable for constructing the evaluation index. Accordingly, a structured questionnaire enquiring about the UD factors was prepared for the method. The validity of this analysis was verified through Kendall's W; the ICC reliability was also verified.

### Modified Delphi Technique

Delphi is a technique that requires the collective judgment of experts in the relevant field. As a consequence, the selection of experts is very important in the Delphi investigation process. Regarding the necessary number of experts participating in the Delphi technique, useful results can be obtained with a small group of 10–15 people (27, 28). A total of 17 field experts were selected as Delphi panels. The Delphi process was implemented over three rounds. In the first round, to incorporate the UD principles in the evaluation criteria, the UD content had to be applied to the evaluation index that was shared with the selected panel by e-mail and was further explained through phone calls or in-person interviews. This enabled multiple UD elements to be applied to the program evaluation index of the gymnasium for the disabled. In addition, it was possible to describe the index items required to operate the program. In the second round of the Delphi process, the results extracted and classified during the first round were shared with the experts by e-mail, and additional opinions to be added or modified in the results were recorded through text analysis. In the third Delphi round, a questionnaire was administered using a 5-point Likert scale to assess the suitability of the evaluation index items to confirm the consensus of the panel on the results extracted during the first and second rounds. To verify the suitability and validity of the evaluation indices, frequency analysis, intraclass correlation coefficient (ICC) reliability analysis, and Kendall's W analysis were conducted using SPSS 21.0. These tests confirmed the degree of agreement among the experts regarding the selections made, and the ranking of each item was analyzed to refine the attributes further.

ICC verification indicates the correlation between the evaluators of the measurement tool. ICC provides relatively stable values when the sample size is small (29). When the reliability index is 0.8 or higher, it shows very high reliability, 0.6 or higher means relatively high reliability, 0.4 or higher refers to moderate reliability, and 0.2 or higher means determined to reasonable reliability (30). Although this index cannot be called absolute, it is often used because it is considered useful (31).

Kendall's W is a method used for checking the degree of agreement among evaluators. It is used when multiple evaluators assess the same subject, and it is measured on an equality scale and a ratio scale. The W value represents a number from 0 to 1, and the closer the value is to 1, the higher the degree of consensus, and the closer it is to 0, the more disagreement there is among the experts (32).

## Results

### UD Factor Reflection Range

UD is a concept created to be applied to architecture or product design, but it was also introduced in learning in 1989 by The National Center for Accessing the General Education Curriculum in the US. The universal design for learning (UDL) was instituted as a new paradigm (33, 34). UDL considers that the difficulty of incorporating the seven UD principles into learning directly and, thus, alternatively provides three core UDL principles (various presentation methods, various expression methods, and various participation methods) to be utilized (35). In this study, the researchers also judged that, as it was, it was too difficult to apply the program to the seven UD principles or the three supplementary principles of PPP to develop program evaluation indicators. Therefore, the expert group selected all seven UD principles and the three supplementary principles that were deemed applicable to the program evaluation index in the first Delphi round. In addition, the UD-based elements constituting the program evaluation index of the sports facility for the disabled were described.

### Selection of Methods for Deriving Evaluation Indicators

Seven studies were selected for creating an evaluation index, and their methods of research and analysis were analyzed. On the basis of this analysis, to derive the results of this study, it was determined that the modified Delphi technique was the most suitable. “Modified Delphi” refers to the case where the content is not structured by the panel, but the researcher uses a structured questionnaire from the beginning (36). The reason for choosing this method was that the UD concept is broad, and the range of differences in interpretation is likely to be large when applied to the sports program aspect. Therefore, it was decided that the Delphi technique should be conducted after first extracting items for the index with the seven UD principles and the three supplementary principles of PPP. Kendall's W was selected as the method of analysis. The same method of analysis was used in a previous study using the Delphi survey, which is known to be the most suitable among the non-parametric tests used with the above-mentioned survey. The closer the W index gets to 1, the stronger the consensus (37).

### Delphi Analysis Results

#### First Delphi

The first Delphi round surveyed the scope of applicability of UD's seven principles and the three supplementary principles to the program evaluation index for the panel and allowed them freedom to describe the essential elements of the Instructor evaluation index. While enquiring about the applicability range of UD's seven principles and three supplementary principles, the first priority of experts was “Product Quality and Aesthetics;” the second priority was the “Perceptible information,” and lastly, they prioritized the “Human environmental consideration.” Detailed results are shown in Table 2.

**Table 2:** Seven principles of UD and three supplementary principle of PPP.

	**Division**	**Contents**
7 Principles	Principle 1: Equitable use	Can anyone use it fairly?
	Principle 2: Flexibility in use	Can you accommodate individual preferences and abilities broadly?
	Principle 3: Simple and intuitive use	Is it easy to understand regardless of users' experience, knowledge, language ability, concentration, etc.?
	Principle 4: Perceptible information	Does it effectively deliver necessary information regardless of users' perceptual ability or surrounding conditions?
	Principle 5: Tolerance For error	Have you minimized the adverse effects and risks of dangerous behavior?
	Principle 6: Low physical effort	Can you minimize fatigue and use it more effectively and safely?
	Principle 7: Size and space for approach and use	Can users provide an appropriate size and space for contacting, reaching, manipulating, and using regardless of their body size and mobility?
3 supplementary principle	Supplementary principle 1: Product quality and aesthetics	The product acts on the user's various five senses, Are you providing the right “feeling”?
	Supplementary principle 2: People's health and the natural environment	From use to disposal of the product, it may be harmful to humans. Isn't the material used?
	Supplementary principle 3: Product durability and production economics	The purchase price or the cost of use is equal to the performance or quality.

In addition, the elements that must be included in the index that evaluates the instructor of the sports facility for the disabled were classified into six evaluation criteria and 22 evaluation indices. The detailed results are shown in Table 3.

**Table 3:** UD application in the scope of evaluation index.

	**Division**	**Frequency (rate)**
7 principles	Principle 1: Equitable use	3 (5.0%)
	Principle 2: Flexibility in use	1 (2.9%)
	Principle 3: Simple and intuitive use	0 (0.0%)
	Principle 4: Perceptible information	6 (9.8%)
	Principle 5: Tolerance for error	2 (3.3%)
	Principle 6: Low physical effort	3 (5.0%)
	Principle 7: Size and space for approach and use	2 (3.3%)
3 supplementary principle	Supplementary principles 1: Product quality and aesthetics	8 (13.3%)
	Supplementary principles 2: People's health and the natural environment	6 (9.8%)
	Supplementary principles 3: Product durability and production economics	3 (5.0%)
Sum total		34 (100%)

#### Second Delphi

In the second round of the Delphi process, the evaluation index was classified into three categories by confirming and revising the results of the first Delphi round, wherein two evaluation indices were deleted. With regards to the second deleted indicator, they explained that the “Priority hiring of professional sports instructors for the disabled” has the same meaning as the indicator “Priority hiring experienced sports instructors for the disabled and those who majored in special sports,” and hence does not need to be considered.

The panel finally selected three principles as suitable to be applied to evaluate sports instructors (Principle 4: Perceptible information, supplementary principles 1: Product Quality and Aesthetics, 2: People's Health and the Natural Environment). This selection was made out of a total of seven UD principles and three supplementary principles. The panel also confirmed that these deletions were reflected in the evaluation index depending on their meaning and intention. Accordingly, the evaluation index was modified during the second Delphi and was further divided into six evaluation criteria according to three categories (selected principles). These six evaluation criteria were then expanded into an evaluation index of 20 items. The evaluation indicators classified as secondary are shown in Table 4.

**Table 4:** Classification of primary assessment criteria and evaluation indicators.

**Assessment basis**	**Evaluation index**
1. Recruitment of professional instructor	1.1 Recruitment of those who have obtained certificates for daily sports and sports for the disabled for the operation of integrated programs
	1.2 Priority recruitment of experienced coaches, special sports majors, and experienced athletes
	1.3 Preferential recruitment of disabled professional athletes
	1.4 Introduction of a professional recruitment system
	1.5 Stabilizing the working environment by securing regular employees
2. Service requirements	2.1 Establishment of working conditions to ensure physical and psychological stability
	2.2 Appropriate personnel per instructor
	2.3 Conducting work based on the Labor Standards Act and the Occupational Safety and Health Act.
	2.4 Creating conditions for focusing on work
3. Performance system	3.1 Establishing a compensation system through performance management
	3.2 Payment of legal allowances in case of overtime or weekend work.
4. Conservative education	4.1 Reinforcement of Instructors' Capabilities Through Conservative Education
	4.2 Providing service and sex crime prevention training to instructors
	4.3 Management and education of instructors through the Sports Association for the Disabled in the relevant local government
5. Support for capacity enhancement	5.1 Support for certificate of sports instructor for the disabled (expenses for education, training hours)
	5.2 Responsibility for the qualification of judges and instructors of persons with disabilities
	5.3 Establishing an education system to enhance instructorship competency
	5.4 Conduct job-related education (sports safety education, first aid treatment, operation event guidance law, etc.)
	5.5 Continued capacity building training to foster experts
6. Strengthen expertise	6.1 Training for integrated disabled/non-disabled sports instructors in the gym
	6.2 Seminar on Sports for the Disabled, Education Support 6.3 Securing superior human resources

#### Third Delphi

In order to verify the validity and reliability of the evaluation indices obtained in the previous round, in the third Delphi round, the panel was asked to respond to a 5-point Likert scale indicating whether the contents of the evaluation index were appropriate. The results are shown in Table 5. To establish the statistical strengths of the evaluation index, we checked the average value and standard deviation value of the index. Data on the quantitative part of the questionnaire can be measured using average scores as a standard measure of the indicator's importance.

**Table 5:** Contents of evaluation index and Results of analysis of reliability, and validity of evaluation indicators.

**Category**	**UD**	**Evaluation conformity**	**Evaluation index**	**Mean**	**SD**	**rank**	**ICC**	**Kendall's W**	**df**	**Result processing**
Recruit	UD 4 PPP 1	1. Recruitment of professional instructors	1.1 Recruitment of those who have obtained certificates for daily sports and sports for the disabled for the operation of integrated programs	4.538	0.508	3.19	0.533	0.144^**^	4	selection
			1.2 Priority recruitment of experienced coaches, special sports majors, and experienced athletes	4.461	0.646	3.04				
			1.3 Introduction of a professional recruitment system	4.538	0.508	3.19				
			1.4 Stabilizing the working environment by securing regular employees	4.615	0.637	1.62				
Education	UD 4 PPP 1 PPP 2	4. Conservative education	2.1 Reinforcement of Instructors' Capabilities Through Conservative Education	4.384	0.752	2.23	0.500	0.154^*^	2	selection
			2.2 Providing service and sex crime prevention training to instructors	4.307	0.617	2.08				
			2.3 Management and education of instructors through the Sports Association for the Disabled in the relevant local government	4.000	0.894	1.69				
		Support for capacity enhancement	5.1 Support for certificate of sports instructor for the disabled (expenses for education, training hours)	4.384	0.752	2.81	0.654	0.177^***^	4	selection
			5.2 Responsibility for the qualification of judges and instructors of persons with disabilities	4.153	0.880	2.27				
			5.3 Establishing an education system to enhance instructorship competency	4.692	0.470	3.35				
			5.4 Conduct job-related education (sports safety education, first aid treatment, operation event guidance law, etc.)	4.692	0.470	3.38				
			5.5 Continued capacity building training to foster experts	4.615	0.496	3.19				
		3. Performance system	3.1 Establishing a compensation system through performance management	4.538	0.646	2.00	0.809	0.259^***^	2	selection
			3.2 Payment of legal allowances in case of overtime or weekend work.	4.307	0.735	1.69				
Welfare	PPP 1	Service requirements	2.1 Establishment of working conditions to ensure physical and psychological stability	4.538	0.760	3.38	0.921	0.137^*^	3	selection
			2.2 Appropriate personnel per instructor	4.461	0.646	2.50				
			2.3 Conducting work based on the Labor Standards Act and the Occupational Safety and Health Act.	4.461	0.646	2.50				
			2.4 Creating conditions for focusing on work	4.615	0.637	2.81				
		Performance system	3.1 Establishing a compensation system through performance management	4.307	0.617	2.19	0.127	0.099	1	remove
			3.2 Payment of legal allowances in case of overtime or weekend work.	4.307	0.928	1.38				

* *p < 0.5*.

** *p < 0.1*.

*** *p < 0.01*.

According to Linstone and Turoff (37), average values can be used to determine whether expert opinions on questionnaire items are consistent and stable (38). Secondly, the ICC index was confirmed by the verification of reliability. Since previous studies have stated that the value of the ICC is valid above 0.2, all items with scores below 0.2 were targeted for removal. Hence, the reliability index of “the performance system” was removed, as it had an ICC value of 0.127. Finally, the results of Kendall's W verification showed that there were statistically significant agreements in five evaluation criteria out of the six. The criteria for evaluation that did not match was the “achievement system.” Accordingly, the “achievement system” of “welfare” was removed, and the remaining five criteria were added.

## Discussion

This study has developed the evaluation indicators for instructor management of sports centers for the disabled following the UD principle. Using Delphi technique we determined which UD elements should be employed and managed by instructors of UD principle-based sports centers for the disabled. Hence, an evaluation index for assessing the suitability of hiring and managing instructors in UD principle-based sports centers for the disabled was created. In this section, we would like to discuss further the meaning of the development of evaluation indicators.

First, the results of a literature survey on UD revealed seven principles and three supplementary rules of commonly used UD. The seven principles were fair use, flexibility in use, simple and intuitive use, recognizable information, tolerance for mistakes, small physical efforts, size, and space for approach and use. The three supplementary rules were the pursuit of quality and psychology, consideration of the human body and the environment, and economic feasibility and validity. In addition, based on previous studies, evaluation indicators were developed through Delphi surveys.

According to previous studies, it can be seen that UD is a necessary element in the present era. In addition, according to UD's definition, it is predicted that it will be applied in various fields (1). Currently, the society we live in is changing into a fast and diverse society (38). In other words, it is important that UD technology, which can be conveniently used together, is applied not only to the hardware field but also to software. Although UD technology has already been applied and actively utilized in various fields other than the field of sports (39–42), it remains to be seen whether it is well applied and operated.

In an environment related to the disabled, even if the centers are good, the impact on the disabled depends on the ability of the instructor (43). Therefore, the management of the instructor who guides the disabled or the evaluation of the instructor's ability should be prioritized (44). In particular, the ability of the instructor is more important for exercise (45). In elite games, the ability of the instructor determines whether the team will win or lose. Similarly, in sports for all, depending on the level of knowledge and various experiences of the instructor, it is possible to continue with fun in sports without injury. A qualified and effective instructor is particularly key for safe and successful exercise for people with disabilities. The disabled have more things to pay attention to than the general public, so expertise in the disabled and experience in guiding the disabled movement is very important.

Therefore, it is necessary to not only select professional instructors but also evaluate them. Various studies related to people with disabilities have also emphasized the importance of evaluation indicators for leadership management (46–51). However, research on the instructor evaluation index of sports centers for the disabled incorporating UD is still insufficient. Therefore, this study would be meaningful to select and analyze questions that fit the instructor management evaluation index through the principle of UD. Moreover, UD will be applied, operated, and expanded in many places throughout our society in the future. Likewise, the practical approach to the development of the instructor evaluation index of sports centers for the disabled based on UD technology is considered an especially important practice.

Second, according to the results of the Delphi survey of the expert group, a large category of evaluation indicators was set for recruitment, education, and welfare. First, recruitment factors appeared to be important in hiring. The evaluation index in recruitment was found to stabilize the working environment by supporting the hiring of people with daily sports and sports certificates for the integrated program operation; establishing preferential recruitment of experienced athletes and special sports majors; introducing a professional recruitment system; and securing regular employees.

This study was conducted based on sports centers. Therefore, it is of paramount importance to select a professional sports instructor. In particular, the expertise of sports for the disabled is noticeable (52). Professionalism includes obtaining certificates, experiences of professional study related to the disabled, and experiences of working in occupations related to the disabled. However, the most important thing is whether someone has direct experience in the field of sports for the disabled. Due to the extent of the differences in the sports-related characteristics of people with disabilities, the instructors with direct experience teaching sports to the disabled must be hired first (8, 53–56).

In addition, stabilization of the working environment is of paramount importance. In the case of Korea, it continues to raise questions about the working environment of sports instructors for the disabled (57). In order to solve the stabilization of the working environment for disabled people, the Ministry of Culture, Sports and Tourism of Korea has increased its budget since 2015. However, the problem of the working environment of sports instructors with disabilities in Korea has not disappeared and is continuously making claims for improvement (57). Likewise, even if a sports facility for the disabled based on the UD principle is completed, the expansion project of these centers will be ruined if the working environment of employees working there is not stabilized. The utilization rate of people who use this place and the disabled among them will be high. One study argued that disabled people in Korea would actively participate in exercise if they had space to exercise near their residence (58). As shown in the study, space is more important than anything else for the disabled, but if there is no one to guide the disabled in the exercise space, it is judged that this is a result of not seeing a distant future. Therefore, stabilizing the working environment of full-time employees is the most important matter.

In terms of education, conservative education, capacity building, and expertise building are considered most important. The evaluation indicators of conservative education are strengthening leadership capabilities through conservative education, service and sex crime prevention education for instructors, and leader education through sports associations for the disabled in local governments. The evaluation indicators for competency building are sports instructor certification support for the disabled, the duty of the disabled to acquire referee and instructor qualifications, the establishment of an education system to strengthen leadership competency, job-related education, and continuous competency building education. Finally, the evaluation indicators for strengthening expertise are UD instructor training education, UD-related seminars in sports centers for the disabled, and educational support.

In certain organizations, groups, organizations, etc., educational support from members is one of the most important parts (59). In particular, job training is very important in the field of exercise instruction. Its importance is further highlighted in the field targeting the disabled (60). In addition, there are also exercises that people with disabilities can do alone, but in most cases, someone has to assist them. Therefore, it is necessary to be accurately aware of sports safety education, first aid education, and operation education for sports. Even if it is a simple method, it is difficult to master it without experiencing it; thus, operation experience is essential.

There is a growing trend of incorporating UD principles into designs for sports centers for the disabled. UD designs may include elements of the space, centers, and equipment that differ from what is generally expected in “traditional” gyms and sports complexes. Reports from regions overseas where UD principles have long been used in construction emphasize that because UD centers are marked differences compared to general centers, the education of leaders and users must be prioritized to maximize the usability of UD centers (61). The recommended education is, above all, related to expertise. Thus, guidelines are needed for sports centers for the disabled incorporating UD, and evaluations of each center's performance should be based on how well they are following those guidelines.

Above all, in sports centers for the disabled, the instructors' service to facility users is important (59, 62). Even with elite disabled athletes familiar with sports, conflicts with instructors continue to occur (63). Therefore, it is paramount that the kindness of instructors is also evaluated. It is of paramount importance for instructors to demonstrate kindness beyond the times when they are providing movement guidance. If these details are included in education and applied to the evaluation index, both the economic level and the social culture of Korea will advance.

Finally, working conditions and performance systems were important in welfare. The evaluation index for working conditions was found to prepare working conditions to ensure physical and psychological stability, arrange appropriate personnel per instructor, conduct tasks based on the Labor Standards Act and the Occupational Safety and Health Act, and create conditions for concentration of assigned departments. The evaluation indicators of the performance system were found to establish a compensation system through performance management, overtime, and payment of legal allowances for weekend work.

Many office workers worldwide value the welfare of their workplace. This value is also paramount to work-life balance (64–66). Additionally, sufficient rest positively affects and increases work efficiency. In the case of Korea, a five-day workweek is operated. However, it is one of the countries with the highest working hours among OECD countries. The happiness index is one of the low countries (67). This means that life in Korea is not happy. For happiness, the welfare of workers should be prioritized, and it should not be forgotten that well-being jobs have a positive impact on performance in the end.

Therefore, even in sports centers for the disabled based on the UD principle, it is important to evaluate the welfare of workers. Also, instructors in sports centers who work with people with disabilities must provide more guidance (and more frequent guidance) to their clients compared to instructors who work with the general public. Work that requires more concentration—and more attention to more details—can generate more work stress for these instructors (59, 62). The heightened level of attention and potential stress for instructors in UD sports centers should be included in the evaluation index, and heightened stress should be evaluated. In addition, careful assessment to determine whether an appropriate compensation system has been implemented was found to be an evaluation point that can clearly motivate workers. When each element of the evaluation items are applied well, investments in UD principle-based sports centers for the disabled will increase, and utilization of the UD sports centers will also be increasingly more positive.

## Conclusion

The purpose of this study was to evaluate and analyze the UD factors related to sports instructors that should be reflected in the assessment and operation of sports centers for the disabled. Using the Delphi technique with experts, we developed evaluation indicators to assess the hiring and training of instructors and measure the instructors' management skills.

The evaluation index developed through the literature review and subsequent rounds of the Delphi techniques consisted of three categories: “recruitment,” “education,” and “welfare.” First, we established the sub-items of the “recruitment” category consisting of one evaluation criterion and four evaluation indices. Supplementary principles, based on the fourth UD principle (recognizable information) and the supplementary principles of PPP (Product Quality and Aesthetics), were also identified as relevant to the study's aims and applied to the evaluation index. Second, we developed the sub-items of the “education” category consisting of three evaluation criteria (remedial education, capacity building, specialization strengthening) and 10 evaluation indices. These were based on the fourth UD principle and the first and second sub-principles of PPP (People's Health and the Natural Environment). The third category, “welfare,” comprises two evaluation criteria (work conditions and achievement system) and six evaluation indices derived from PPP's first supplement. The chosen welfare category items were also applied to the evaluation indices.

Based on our findings and the evaluation indices, the recommendations of this study are as follows: First, the work of validating the evaluation indices should be carried out by applying the developed evaluation index to instructors and operators of public sports centers and sports centers for the disabled that offer integrated programs. Second, more evaluation indicators are needed (and should be developed) for other functions—such as programs, public relations, safety, and finance—of sports centers for the disabled based on the UD principles. An awareness education program promoting and clarifying the UD concept and informing the public about the purpose of the centers is needed to ensure the provision of a fair and comfortable environment wherein everyone has access to UD sports centers. Moreover, steps should be taken to guarantee that everyone can easily and conveniently patronize the centers.

## Data Availability Statement

The original contributions presented in the study are included in the article/supplementary material, further inquiries can be directed to the corresponding author/s.

## Ethics Statement

Written informed consent was obtained from the individual(s) for the publication of any potentially identifiable images or data included in this article.

## Author Contributions

EY and AO: original draft preparation. AO: data analysis. EY and JS: critical review of the contents. AO and JS: data collection and critical review of the manuscript. AO, JS, and EY: supervision. All authors have read and agreed to the published version of the manuscript.

## Funding

This research project was supported by the Sports Promotion Fund of Seoul Olympic Sports Promotion Foundation from Ministry of Culture, Sports, and Tourism of the Republic of Korea (R&D/1375026989).

## Conflict of Interest

The authors declare that the research was conducted in the absence of any commercial or financial relationships that could be construed as a potential conflict of interest.

## Publisher's Note

All claims expressed in this article are solely those of the authors and do not necessarily represent those of their affiliated organizations, or those of the publisher, the editors and the reviewers. Any product that may be evaluated in this article, or claim that may be made by its manufacturer, is not guaranteed or endorsed by the publisher.
